# Prediction of exposure-driven myelotoxicity of continuous infusion 5-fluorouracil by a semi-physiological pharmacokinetic–pharmacodynamic model in gastrointestinal cancer patients

**DOI:** 10.1007/s00280-019-04028-5

**Published:** 2020-03-09

**Authors:** Usman Arshad, Su-arpa Ploylearmsaeng, Mats O. Karlsson, Oxana Doroshyenko, Dorothee Langer, Edgar Schömig, Sabine Kunze, Semih A. Güner, Roman Skripnichenko, Sami Ullah, Ulrich Jaehde, Uwe Fuhr, Alexander Jetter, Max Taubert

**Affiliations:** 1 Faculty of Medicine and University Hospital Cologne, Center for Pharmacology, Department I of Pharmacology, University of Cologne, Cologne, Germany; 2 Department of Pharmaceutical Biosciences, Uppsala University, Uppsala, Sweden; 3 Department of Radiotherapy, University Hospital Cologne, Cologne, Germany; 4 Institute of Pharmacy, Clinical Pharmacy, University of Bonn, Bonn, Germany; 5 Department of Clinical Pharmacology and Toxicology, University Hospital Zurich, University of Zurich, Zurich, Switzerland

**Keywords:** 5-Fluorouracil, Pharmacokinetics, Pharmacodynamics, Pharmacogenetics, Myelosuppression

## Abstract

**Purpose:**

To describe 5-fluorouracil (5FU) pharmacokinetics, myelotoxicity and respective covariates using a simultaneous nonlinear mixed effect modelling approach.

**Methods:**

Thirty patients with gastrointestinal cancer received 5FU 650 or 1000 mg/m^2^/day as 5-day continuous venous infusion (14 of whom also received cisplatin 20 mg/m^2^/day). 5FU and 5-fluoro-5,6-dihydrouracil (5FUH2) plasma concentrations were described by a pharmacokinetic model using NONMEM. Absolute leukocyte counts were described by a semi-mechanistic myelosuppression model. Covariate relationships were evaluated to explain the possible sources of variability in 5FU pharmacokinetics and pharmacodynamics.

**Results:**

Total clearance of 5FU correlated with body surface area (BSA). Population estimate for total clearance was 249 L/h. Clearances of 5FU and 5FUH2 fractionally changed by 77%/m^2^ difference from the median BSA. 5FU central and peripheral volumes of distribution were 5.56 L and 28.5 L, respectively. Estimated 5FUH2 clearance and volume of distribution were 121 L/h and 96.7 L, respectively. Baseline leukocyte count of 6.86 × 10^9^/L, as well as mean leukocyte transit time of 281 h accounting for time delay between proliferating and circulating cells, was estimated. The relationship between 5FU plasma concentrations and absolute leukocyte count was found to be linear. A higher degree of myelosuppression was attributed to combination therapy (slope = 2.82 L/mg) with cisplatin as compared to 5FU monotherapy (slope = 1.17 L/mg).

**Conclusions:**

BSA should be taken into account for predicting 5FU exposure. Myelosuppression was influenced by 5FU exposure and concomitant administration of cisplatin.

**Electronic supplementary material:**

The online version of this article (10.1007/s00280-019-04028-5) contains supplementary material, which is available to authorized users.

## Introduction

The pyrimidine antimetabolite 5-fluorouracil (5FU) is being used since decades for the treatment of gastrointestinal solid malignancies [1]. Dose, but also route and schedule of administration have been identified to influence 5FU pharmacokinetics (PK) and effects [2, 3]. Considerable variations in PK and toxicity are associated with a given 5FU dosing regimen [4]. Investigations have been carried out evaluating patient’s factors to predict 5FU exposure, where a relationship between body surface area (BSA) and 5FU clearance (CL_5FU_) was reported [3, 5]. CL_5FU_ was found to be lower in females [6] and at older age [5, 7]. Due to saturable hepatic degradation, 5FU PK is considered to be non-linear in nature [8]. Additionally, elimination of the drug was reported to be influenced by hepatic metastases [3] and by glomerular filtration rate as measured by creatinine clearance [6].

Being a prodrug, 5FU requires enzymatic activation. A small fraction of an administered dose is metabolised into cytotoxic nucleotides, while most of the drug is degraded to 5-fluoro-5,6-dihydrouracil (5FUH2) mainly by hepatic dihydropyrimidine dehydrogenase (DPD) [9]. Some rare variants of the highly polymorphic DPD gene (*DPYD)* are responsible for complete or partial loss of DPD activity, which is related to increased 5FU toxicity [10]. Belonging to the class of antimetabolites, 5FU inhibits thymidylate synthase (TS), ultimately leading to the impairment of DNA synthesis [11]. Polymorphisms in the gene encoding TS influence toxicity and response of 5FU-based therapeutic regimens [12]. Methylenetetrahydrofolate reductase (MTHFR) is involved in the formation of the reduced folate cofactor, which is required for the inhibition of TS. Genetic polymorphisms in the gene encoding MTHFR are associated with altered enzymatic activity, thereby influencing sensitivity towards 5FU [13].

5FU is commonly used in combination with other antineoplastic drugs and with radiotherapy. A therapeutic regimen known as FOLFIRINOX including 5FU, folinic acid, oxaliplatin and irinotecan is frequently employed for the treatment of colorectal and pancreatic cancer [14]. Another regimen, called de Gramont, includes a combination of 5FU and leucovorin (folinic acid) and has been reported to possess low-toxicity profile, increased response rate and progression-free survival [15]. For oesophageal cancer, combination with cisplatin is one of the recommend drug treatments [16]. Approximately 10–30% of 5FU-treated patients experience severe treatment-related toxicity [17], where myelosuppression and mucositis have been reported as main dose limiting side effects in 5FU treatment [18]. Continuous infusions exhibit lower myelosuppression with greater efficacy and are considered superior over the bolus administrations [19]. Furthermore, 5FU has a narrow therapeutic index with severe toxicities tending to occur with AUC values > 25 mg h/L during continuous venous infusion [20]. Therefore, therapeutic drug monitoring is considered valuable to achieve optimal 5FU exposure with minimal serious toxicity [21].

Semiphysiological myelosuppression models were developed in both animals and human beings to understand time course and extent of leukopenia following administration of cytotoxic antineoplastic drugs, thus facilitating the drug development and therapy [22]. Models incorporating white blood cell (WBC) count over time are helpful to predict the time (*T*_nadir_) and depth (WBC_nadir_) of lowest total WBC count and the duration of the recovery period to administer the next cycle of a regimen [23]. Efforts have been made to predict the time course of myelosuppression by 5FU in rats [24]. Population analysis was carried out for the hematological toxicity in breast cancer patients treated with combined 5FU, epirubicin and cyclophosphamide regimen [25] but such a study with 5FU monotherapy is lacking.

The objective of the present study was to describe the PK and associated variability of 5FU and its metabolite by developing an empirical model. Subsequently, it was aimed to establish the relationship between 5FU exposure and myelotoxicity through a semi-mechanistic PKPD model. The study was further focused towards the identification and quantitative description of covariates on 5FU PK and myelotoxicity, especially patient demographics and genotypes (*DPYD*, *MTHFR*, *TS*).

## Methods

### Patients and treatment plan

The study was approved by the Ethics Committee of the Medical Faculty of the University of Cologne, Germany (application number 02-171), and was conducted according to the Declaration of Helsinki and national and international legal stipulations and guidelines in 2002–2005 [26, 27]. Sample size was estimated using WinBiAS (version 7.01, epsilon Verlag, Darmstadt, Germany) considering interindividual variability (IIV) in 5FU pharmacokinetics to be at least 30%. To assess the effect of a covariate on 5FU pharmacokinetics, assuming a linear coefficient of correlation *ρ* = 0.5477 (explaining a fraction of *ρ*^2^ = 0.3 of variability) with a power of 90% and *α* = 0.05, *n* = 30 patients were required. To account for possible dropouts, 33 patients with colorectal or oesophageal cancer were planned to be enrolled in the study after the provision of written informed consent. Eligibility criteria included age ≥ 18 years; Karnofsky performance status ≥ 70%; life expectancy ≥ 3 months; adequate haematopoietic, hepatic, and renal function. Exclusion criteria were prior chemotherapy or radiotherapy, and concomitant drugs (not included in the chemotherapeutic regimen) known to interfere with 5FU PK and/or pharmacodynamics. The patients with colorectal cancer received 5FU 650 or 1000 mg/m^2^/day as 24-h continuous venous infusion for 5 days, and radiotherapy. The patients with oesophageal cancer additionally obtained cisplatin 20 mg/m^2^/day for 5 days before, or together with 5FU administration. Any decision on treatment was made according to the clinical situation and was not influenced by the study. WBC count was evaluated once prior to and 1–3 times per week after 5FU administration, but prior to the second cycle starting on day 28, as the assessment of myelosuppression was aimed to be investigated under the influence of single cycle of treatment. Only the first cycle was monitored in each participant. Covariate data regarding patient demographics and essential laboratory values were collected prior to the treatment.

### Genotyping

DNA were extracted from peripheral blood using the QIAamp DNA Blood Mini Kit (Qiagen, Hilden, Germany). For *DPYD* genotyping [28], PCR amplification of all 23 coding exons and exon–intron boundaries of the *DPYD* gene was carried out. PCR products were separated on 1.6% agarose gels, visualized with ethidium bromide and purified using a QIA quick Gel Extraction Kit (Qiagen, Hilden, Germany). Samples were sequenced on an ABI 3100 automated DNA sequencer (Applied Biosystems, Foster City, CA, USA). TS genotyping was carried by PCR amplification [29] of the *TS* promoter enhancer region containing the double and triple tandem repeats using the following primers: forward 5′AAAAGGCGCGCGGAAGGGGTCCT3′; reverse 5′TCCGAGCCGGCCACAGGCAT3′. A total of 32 cycles (94 °C for 40 s, 62 °C for 40 s and 72 °C for 1 min) and extension at 72 °C for 5 min were carried out following hot start at 94 °C for 4 min. The PCR product was analysed in a 3% agarose gel. The triple repeat (3R/3R) had a 144 bp PCR product, the double repeat (2R/2R) a 116 bp product. For *MTHFR* genotyping [30], the PCR used forward primer 5′TGAAGGAGAAGGTGTCTGCGGGA3′ and reverse primer 5′AGGACGGTGCGCTGAGAGTG3′. Restriction fragment analysis was carried out using *Hinf* I (Fermentas, St. Leon-Rot, Germany). The C → T substitution at nucleotide 667 creates a *Hinf* I digestion site resulting in two fragments (175 bp and 23 bp) of the PCR product.

### Analysis of 5FU and 5FUH2 plasma concentrations

Analytical-grade reagents were purchased from Merck (Darmstadt, Germany). 5FU (Sigma, St. Louis, MO, USA) was purchased as crystalline form, pure > 95%, 5FUH2 (26.5% pure) was supplied by Syncom (Groningen, The Netherlands), and 5-chlorouracil (5-CU), the internal standard, was obtained from Arcos Organics (Geel, Belgium).

Blood samples (4.5 mL each) were withdrawn during the first cycle using Li^+^-heparinized tubes pre-dose, 36, 48, and 108 h after the start of 5FU infusion, at the end of infusion, and 5, 30, 60 and 90 min thereafter. The samples were immediately placed in an ice water bath and centrifuged at + 4 °C. Plasma was stored at − 80 °C until analysis. 5FU and 5FUH2 in plasma were quantified by reverse-phase HPLC method with UV detection. Briefly, 0.7 mL of plasma was mixed with 20 µL of 100 µg/mL 5-CU (internal standard) and extracted with 7 mL of isopropanol/ethyl acetate (5:95, v/v). Samples were mixed and centrifuged (3500 rpm, 10 min) to separate the organic phase, which was evaporated to dryness. The samples were reconstituted with 100 µL of 50 mM K_2_HPO_4_ (pH 4.0), and 40 μL was injected into the HPLC system. 5FU and 5FUH2 were separated on an Ultrasphere ODS C_18_ column (5 μm, 250 × 4.6 mm, Beckman Coulter, Brea, CA, USA). Elution was performed under gradient condition as follows: 50 mM K_2_HPO_4_ (A) for 17 min, acetonitrile (B) 0–50% over 1 min and maintained at 50% for 5 min; initial conditions were restored by decreasing B to 0% over 1 min, and the column was equilibrated with 100% A for 5 min. The chromatographic instrument was a Waters 2690 Separations Module (Waters, Milford, MA, USA) with a Waters 996 photodiode array detector. Detection of 5FU, 5FUH2 and 5-CU were carried out at 265, 220 and 270 nm, respectively. Data analysis was performed by the Millennium 2.1 software (Waters, Milford, MA, USA). The LLOQs were 0.005 and 0.01 µg/mL for 5FU and 5FUH2, respectively. The 5FU and 5FUH2 intra-assay coefficients of variation ranged from 0.34 to 7.15% and from 0.53 to 2.76%, respectively, while the inter-assay coefficients of variation for a 5-day validation were 0.1–3.4% and 2.3–9.0%, respectively.

### Data analysis

#### Model development and selection criteria

R (version 3.2.3) was used for data manipulation and exploratory evaluation [31]. Population parameter estimates were obtained using first-order conditional estimation with interaction (FOCE-I) algorithm in NONMEM 7.4.2 (ICON, Development Solutions, Elliot City, MD, USA) [32]. Model development process was aided by Perl-speaks-NONMEM (PsN) toolkit (Version 4.7.0) [33]. IIV in model parameters was estimated assuming a log-normal distribution. Additive, proportional and combined error models were implemented to estimate residual unexplained variabilities (RUV) for 5FU and 5FUH2. Model selection/rejection was guided by a decrease in objective function value (OFV) which was assumed to be chi-squared distributed (*p* < 0.05, corresponding to a ΔOFV ≥ 3.84 given a change by one degree of freedom), diagnostic plots, scientific plausibility and precision of parameter estimates. Precision of population parameter estimates was assessed with the help of a bootstrap procedure using 1000 sample replicates.

#### Pharmacokinetic analysis

An empirical compartmental model for 5FU was first developed and then expanded to a combined model incorporating 5FUH2 data. The drug was presumed to be eliminated from the central compartment where elimination was tested to follow either a linear behaviour or nonlinear Michaelis–Menten kinetics. The fraction of 5FU converted to 5FUH2 was fixed a priori to 0.85, according to the literature [6, 34]. PK parameters were estimated based on the absolute dose administered.

#### Pharmacodynamic analysis

The PD model was developed according to Friberg et al. [24, 34] using simultaneous approach. The model was driven by 5FU plasma concentrations from the PK model and comprised a compartment of proliferating leukocytes (rate constant describing the proliferation of cells: *k*_prol_), transit compartments representing leukocytes undergoing maturation (rate constant describing the transfer between transit compartments: *k*_tr_) and a compartment of circulating leukocytes (rate constant describing the rate of exit from the circulating compartment: *k*_circ_). Parameters were the baseline circulating leukocyte count (Circ_0_) representing the number of cells prior to 5FU administration, mean transit time (MTT = [*n* + 1]/*k*_tr,_ where *n* denotes the number of transit compartments) and a parameter *γ* describing a negative feedback of circulating cells on the rate of self‐renewal of the proliferative cells (feedback = [Circ_0_/Circ]^*γ*^). The number of parameters to be estimated were minimized by assuming *k*_prol_ = *k*_tr_ = *k*_circ_. 5FU plasma concentrations were assumed to inhibit the proliferation of leukocytes.

The drug effect (*E*_drug_) on proliferating cells was assumed to be driven by individual predicted 5FU plasma concentrations (*C*_p_) and was incorporated into the model as *k*_prol_ × (1 − *E*_drug_). *E*_drug_ was either formulated as a linear model (*E*_drug_ = slope × *C*_p_) or a nonlinear model (*E*_drug_ = *E*_max_ × *C*_p_/(EC_50_ + *C*_p_)).

#### Covariate analysis

Covariates tested in the PK analysis included demographics (age, weight, height, sex, body mass index, lean body weight, BSA); predose plasma concentrations of alanine aminotransferase (ALT), aspartate aminotransferase (AST) and γ-glutamyltransferase (γ-GT), and albumin; and *DPYD, TS* and *MTHFR* genotypes. The effect of co-medication with cisplatin was tested on the slope of the linear effect model. Scientific plausibility was the primary basis for covariate pre-selection, while graphical evaluation (residuals and individual PK estimates versus covariates) was performed to assist the inclusion decision. A comparative analysis was carried out to assess any possible superiority of other indices representing body mass over the BSA such as BMI, LBW and allometric scaling with body weight.

#### Simulation design

The effect of concomitant cisplatin administration on leukocyte suppression was evaluated. WBC counts over time were simulated for virtual subjects receiving a 5-day continuous infusion with and without cisplatin co-medication. WBC_nadir_ and *T*_nadir_ for the respective regimens were determined to assess the degree of myelosuppression.

Another simulation scenario aimed towards the comparative assessment of the time course of myelosuppression theoretically produced by the 5FU component contained in a single cycle of the two standard dosage regimens used in current clinical practice; to this end, the effects of the other components of the regimens were ignored. The standard FOLFIRINOX regimen combines oxaliplatin (85 mg/m^2^ over 2 h) with folinic acid (200 mg/m^2^) followed by irinotecan (180 mg/m^2^ over 90 min) and 5FU (400 mg/m^2^ bolus) followed by 2400 mg/m^2^ 5FU over 46 h, all on day 1 and repeated every 2 weeks [14]. The de Gramont regimen is described as follows: high-dose folinic acid (200 mg/m^2^) followed by 5FU i.v. bolus (300 mg/m^2^) and continuous infusion (300 mg/m^2^) on days 1, 2, 14 and 15, repeated every 4 weeks. In the absence of toxicity, 5-FU is increased to 400 mg/m^2^ i.v. bolus and continuous infusion at course 2 and to 500 mg/m^2^ at course 3 and from course 4 maintained at 500 mg/m^2^ [15]. Simulated WBC_nadir_ and *T*_nadir_ were observed for the treatment with FOLFIRINOX (400 mg/m^2^ bolus 5FU followed by 2400 mg/m^2^ 5FU over 46 h) and de Gramont (5FU 300 mg/m^2^ i.v. bolus followed by 300 mg/m^2^ continuous infusion over 24 h) regimens.

## Results

### Patient characteristics

Thirty-three patients were included in the study; of these, three patients dropped out prior to the first administration of 5-FU. The remaining 30 patients who all completed the study comprised 5 women and 25 men with an age ranging between 37 and 73 years. 16 patients with colorectal cancer were administered 5FU only, while 14 with oesophageal cancer were treated with the combination of 5FU with cisplatin. Patient demographics, primary tumor location, pre-treatment values of haematology and clinical chemistry parameters are summarized in Table 1.

**Table 1:** Patient characteristics (*n* = 30)

Characteristics	Value
Sex (*n* male/ *n* female)	25/5
Median age, years (range)	59.5 (37–73)
Median Karnofsky performance status (range)	100% (100–100)
Tumour primary site (*n*)	
Oesophagus	14
Rectal	2
Colorectal	13
Anus	1
Median body height, m (range)	1.75 (1.61–1.86)
Median body weight, kg (range)	76 (46–111)
Median BMI, kg/m^2^ (range)	24.2 (16.9–33.2)
Median BSA, m^2^ (range)	1.95 (1.48–2.33)
Median baseline laboratory values (range)	
Haemoglobin (g/dL)	13.7 (10.1–16.6)
Platelet count (× 10^3^/µL)	277 (48–426)
Erythrocyte count (× 10^6^/µL)	4.6 (3.8–5.5)
Leukocyte count (× 10^9^/L)	6.90 (4.68–11.28)
Plasma albumin (g/dL)	42 (35–47)
Plasma ASAT (U/L)	18 (9–50)
Plasma ALAT (U/L)	15 (8–90)
Plasma γ-GT (U/L)	24 (13–81)
Plasma total bilirubin (mg/dL)	0.45 (0.4–0.5)
Plasma creatinine (mg/dL)	0.85 (0.44–1.06)
Co-medication with cisplatin (*n*)	14

*BSA* body surface area, *BMI* body mass index, *ASAT* aspartate aminotransferase, *ALAT* alanine aminotransferase, *γ-GT* gamma-glutamyl transferase

### Genotypes

Analysis of the *DPYD* gene revealed the presence of 6 polymorphisms in 22 of 30 patients. Eight patients had multiple mutations in the coding region of the *DPYD* gene (Table 2). None of the known rare (< 2%) mutations causing extremely reduced or absent DPD activity, such as exon 14 (DPYD*2A) G>A skipping mutation [35], was found in the study population. The six *DPYD* polymorphisms detected in our study were considered to result in either normal (i.e. 1236G>A [36]) or partially reduced enzyme activity [37].

**Table 2:** Allele frequencies of polymorphisms in the *DPYD* gene found in patients

Polymorphisms in the *DPYD* gene		Effect (nucleotide change)	Wild type (*n*)	Heterozygous mutant (*n*)	Homozygous mutant (*n*)	Allelic frequency (%)
^a^*DPYD* nomenclature	Exon					
DPYD*9A	2	Cys29Arg (85T>C)	20	8	2	12/60 (20%)
–	6	Met166Val (496A>G)	22	8	0	8/60 (13.33%)
–	11	Glu412Glu (1236G>A)	29	1	0	1/60 (1.67%)
DPYD*4	13	Ser534Asn (1601G>A)	29	1	0	1/60 (1.67%)
DPYD*5	13	Ile543Val (1627A>G)	19	9	2	13/60 (21.67%)
DPYD*6	18	Val732Ile (2194G>A)	28	2	0	2/60 (3.33%)

*DPYD* dihydropyrimidine dehydrogenase gene

^a^Source of nomenclature: Mcleod et al*.* [44]

With regard to the *TS* genotype, 5 (16.7%) patients were homozygous for the triple repeat (3R/3R), 19 (63.3%) were heterozygous (2R/3R), and 6 (20%) were homozygous (2R/2R) for the double repeat variant within the *TS* promoter region. As for C677T *MTHFR* genotype, 13 of 30 patients (43.3%) were CC (wild type), 12 (40%)—CT (heterozygous mutant), and 5 (16.7%)—TT (homozygous mutant).

### Pharmacokinetic model

**Fig. 1:**
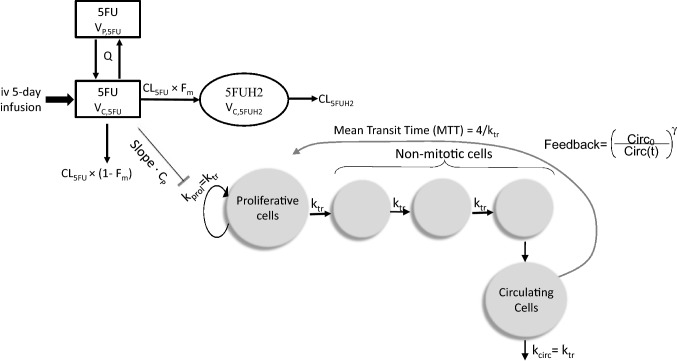
Schematic representation of PKPD model. Compartments with white background reflect the PK model describing 5FU and 5FUH2 plasma concentrations, while those with grey background reflect the PD model describing total WBC count over time. *k*_*prol*_ first order rate constant of proliferation, *k*_*tr*_ first-order rate constant of transit, *k*_*circ*_ first-order rate constant of elimination of circulating cells, *Circ*_*0*_ baseline leucocyte count, *γ* feedback parameter, *C*_*p*_ 5FU plasma concentration, *V*_*C,5FU*_ 5FU central volume of distribution, *V*_*P,5FU*_ 5FU peripheral volume of distribution, *CL*_*5FU*_ 5FU total clearance, *Q* intercompartmental clearance, *F*_*m*_ fraction of 5FU converted to 5FUH2, *V*_*C,5FUH2*_ 5FUH2 central volume of distribution, *CL*_*5FUH2*_ 5FUH2 clearance, drug effect: *E*_drug_ = slope·*C*_p_

**Fig. 2:**
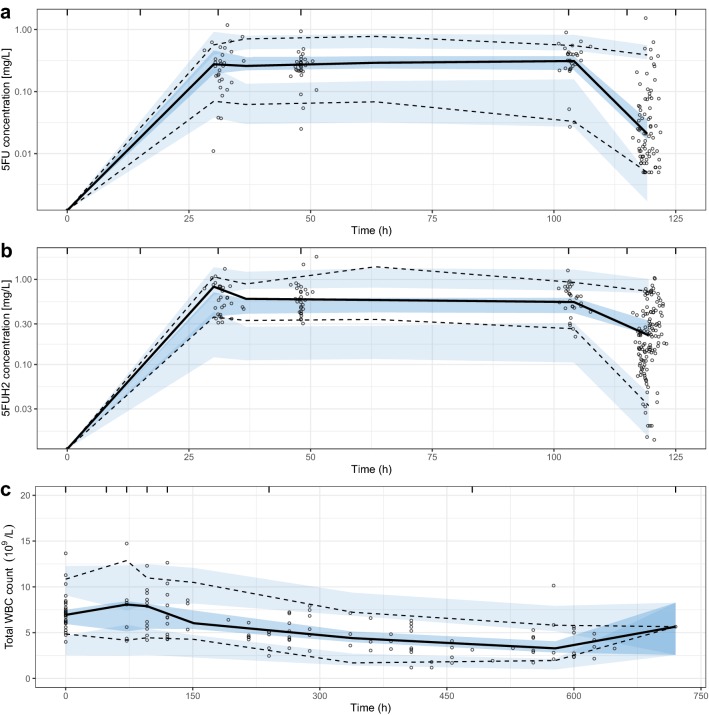
Visual predictive checks for 5FU (**a**) and 5FUH2 (**b**) plasma concentration data and total WBC count (**c**) over time. Continuous and dashed lines represent median, 2.5th and 97.5th percentiles of the observed data. Shaded areas are the 95% confidence interval for median, 2.5th and 97.5th percentiles of the simulated data. 5FU and 5FUH2 plasma concentrations are presented on a log scale

199 and 251 quantifiable plasma concentrations of 5FU and 5FUH2, respectively, were part of the pharmacokinetic model development. Figure 1 provides a schematic representation of the PKPD model. A two-compartment model with linear elimination (∆OFV of 210 compared to one-compartment model) best described the 5FU concentration–time data, and a one-compartment model was appropriate for the 5FUH2 data. IIV in the combined model was included for CL_5FU_, CL_5FUH2_, *V*_C,5FU_ and *V*_C,5FUH2_. IIV on *V*_P,5FU_ was removed because of a high shrinkage value, while IIV on intercompartmental clearance (*Q*) was negligible and hence removed from the final model. A proportional error model was appropriate to model RUV for both 5FU and 5FUH2. Visual predictive checks (VPCs) indicated an adequate prediction of the 5FU and 5FUH2 concentrations by the model (Fig. 2). Population pharmacokinetic parameter estimates are presented in Table 3.

**Table 3:** Population pharmacokinetic and pharmacodynamic parameter estimates

Parameter	NONMEM estimates		NONMEM RSE (%)	Bootstrap estimates		Bootstrap RSE (%)	95% CI
5FU							
CL_5FU_ (L/h)	256	5.47		249	6.36		224–276
*V*_C,5FU_ (L)	5.85	39.3		5.56	42.0		2.41–9.87
*V*_P,5FU_ (L)	24.0	22.9		28.5	81.5		13.3–59.3
*Q* (L/h)	17.3	30.7		14.8	29.8		9.66–23.4
BSA effect (m^−2^)^a^	0.71	23.5		0.77	28.0		0.44–1.14
AUC_24,5FU_ (mg h/L)^b^	6.72	–		6.72	–		4.76–8.74
5FUH2							
*F*_m_ (%)	85	–		85	–		Fixed
CL_5FUH2_ (L/h)	124	6.61		121	7.11		108–136
*V*_C,5FUH2_ (L)	100	13.0		96.7	14.37		74.8–119
AUC_24,5FUH2_ (mg h/L)^b^	12.2	–		12.2	–		7.12—19.2
Total WBC count							
CIRC_0_ (× 10^9^/L)	7.16	5.23		6.86	4.50		6.38–7.37
MTT (h)	261	6.70		281	13.1		224–344
Slope_comb_ (L/mg)	2.10	18.9		2.82	27.2		1.53–3.77
Slope_mono_ (L/mg)	1.31	44.2		1.17	25.0		0.78–1.72
*γ*	0.17	–		0.17	–		Fixed
IIV (%CV)							
CL_5FU_	24.9	17.2		23.0	43.1		12.3–30.1
*V*_C,5FU_	130	45.4		145	57.0		75.3–204
CL_5FUH2_	30.5	27.1		28.9	28.0		21.8–35.1
*V*_C,5FUH2_	58.9	62.7		59.6	76.8		30.7–96.3
CIRC_0_	16.8	69.6		16.4	52.0		8.29–22.8
RUV (*σ*2)							
Proportional error 5FU	0.36	10.2		0.32	9.37		0.23–0.44
Proportional error 5FUH2	0.14	8.06		0.14	9.61		0.10–0.18
Proportional error total WBC count	0.08	8.70		0.08	8.97		0.06–0.11

*RSE* relative standard error, *CI* confidence interval, *CL*_*5FU*_ total clearance of 5FU, *V*_*C,5FU*_ 5FU central volume of distribution, *V*_*P,5FU*_ 5FU peripheral volume of distribution, *Q* intercompartmental clearance, *BSA* body surface area, *F*_*m*_ fraction of 5FU converted to 5FUH2, *CL*_*5FUH2*_ clearance of 5FUH2, *CIRC*_*0*_ baseline leukocyte count, *MTT* mean transit time, *Slope*_*comb*_ slope parameter for combination therapy with cisplatin, *Slope*_*mono*_ slope parameter for 5FU monotherapy, The “Slope” parameter represents the relationship between effect and drug concentration into bone marrow (*E*_drug_ = slope × *C*_p_), *C*_p_ plasma concentration, *IIV* interindividual variability, *RUV* residual unexplained variability, *CV* coefficient of variation

^a^Fractional change in CL per m^2^ difference from median BSA value

^b^Calculated by obtaining time integral of drug concentrations using an additional compartment in NONMEM

### Pharmacodynamic model

In total, 135 observations for total WBC count were available for 29 patients. None of the patients received a WBC count-modifying drug (e.g. filgrastim). The semi-mechanistic model with three transit compartments adequately described the time course of myelosuppression (Fig. 2). A linear model was preferred over an *E*_max_ model, as the *E*_max_ model did not provide any additional goodness-of-fit to describe the PK/PD relationship. The estimated parameter *γ* for the feedback mechanism was inconsistent across different runs and was, therefore, fixed to a value of 0.17 according to the available literature [22]. Fixing the parameter estimate for *γ* did not have a significant impact on the model fit (∆OFV = 3.68). IIV on MTT and slope were not kept in the final model as they displayed high shrinkage and provided no further improvement with ∆OFV values of 1.32 and 0.09, respectively. A proportional error model was found adequate to model RUV. Pharmacodynamic parameter estimates are presented in Table 3.

### Covariate relationships

Estimated IIV in CL_5FU_ in the covariate-free model was 32.8% (CV), whereas a 9% reduction in IIV resulted from the inclusion of BSA as covariate. Scaling with BSA (∆OFV = 10.1) was found superior to that with LBW (∆OFV = 6.4), BMI (∆OFV = 4.3) and body weight (∆OFV = 5.0). CL_5FU_ point estimates were 263 L/h [231–295] and 175 L/h [87–263] in patients with wild-type *DPYD* genotype and homozygous mutations, respectively. Precision of these estimates was poor, and the effect of *DPYD* was not statistically significant, probably due to small number of mutations in the *DPYD* gene found in the studied population. IIV of CL_5FU_ was correlated to *MTHFR* genotype with a ∆OFV of 8.98, but the covariate effect was not included in the model because of limitations with regard to mechanistic plausibility. Scaling CL_5FUH2_ with individual BSA reduced OFV by 6.76 points with a marginal reduction in IIV (~ 2.1%). The estimate for BSA effect on CL_5FUH2_ (0.73 m^−2^) was close to that on CL_5FU_ (0.79 m^−2^); therefore, the BSA effect was included as a single parameter in the final model assuming a similarity in disposition kinetics between 5FU and 5FUH2. Using single parameter instead of two separate parameters provided no significant change in model fit (∆OFV = 0.35). Inclusion of cisplatin co-medication as a covariate upon slope parameter provided an improvement in model fit by reduction of 18.6 OFV points. Thus, the covariate relationship part of the PKPD model included effects of BSA on CL_5FU_ and CL_5FUH2_, and of cisplatin comedication on slope.

Bootstrap analysis using the final PKPD model including the covariate relationships resulted in 681 runs with successful minimization, 318 runs with rounding errors, whereas only a single run failed during the execution. Parameter estimates obtained from bootstrapping were very close to NONMEM estimates (Table 3).

### Simulated total WBC count over time

**Fig. 3:**
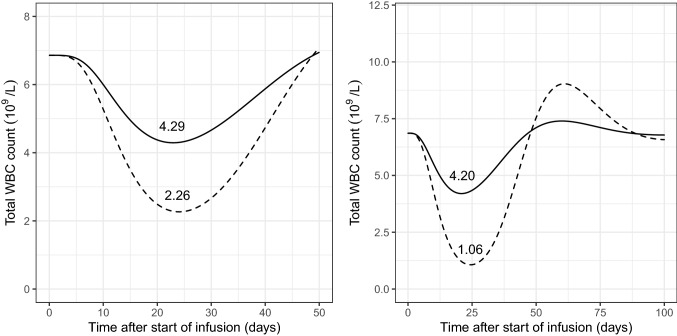
Left panel; simulated total WBC count over time. Continuous line represents individuals receiving 5FU monotherapy. Dashed line represents individuals receiving combination therapy with 5FU and cisplatin. Numbers represent corresponding WBC_nadir_ values. Right panel: simulated total WBC over time for effects attributable to a 5FU dose as used in the FOLFIRINOX (400 mg/m^2^ bolus 5FU followed by 2400 mg/m^2^ over 46 h) versus the de Gramont regimens (300 mg/m^2^ i.v. bolus followed by 300 mg/m^2^ continuous infusion over 24 h): Continuous lines represent an individual receiving 5FU according to de Gramont regimen, while dashed lines represent an individual receiving the dose according to FOLFIRINOX regimen. Effects apply for a single treatment course, and those of the other components of the respective regimens are ignored in this figure. Numbers represent corresponding WBC_nadir_ values

Differences in simulated WBC count over time for 5FU monotherapy (5FU_mono_) and combination therapy (5FU_comb_) are presented in Fig. 3 (left panel). A higher degree of myelosuppression was observed for the typical individual receiving 5FU_comb_ in comparison to the individual receiving 5FU_mono_. Simulated temporal changes in total WBC count (Fig. 3, right panel) showed a higher degree of myelosuppression for virtual subjects administered with the higher 5FU exposure in FOLFIRINOX regimen in comparison to de Gramont regimen.

## Discussion

A semi-physiological PK/PD model of 5FU during continuous venous infusion was developed. Covariate effects including genetic variants of the main enzymes involved in 5FU PK and myelosuppression were tested. BSA was identified as a factor significantly influencing 5FU pharmacokinetics. Cisplatin co-administration was found to aggravate myelotoxicity. The current investigations are of particular value because they establish a link between 5FU PK and myelosuppression in the same patients, where we were also able to characterize the PD interaction between 5FU and cisplatin.

Approaches adapted to describe pharmacokinetics of 5FU have been nicely summarized by Deyme et al. [14]. In most of the cases, a two-compartment model was found adequate to describe 5FU PK [3, 37–39], while some studies presented a one-compartment model [6, 40]. Most of these studies demonstrated a linear elimination [6, 37, 41], whereas a nonlinear elimination was also observed occasionally [3, 39]. Both linear and nonlinear elimination kinetics were reported in one case [39]. In the current evaluation, a two-compartment model with linear elimination was the best to describe 5-FU PK. CL_5FU_ of 249 L/h was comparable to the estimates obtained in similarly designed studies. Non-compartmental analysis with a 5-day continuous infusion estimated the CL_5FU_ to be 257 L/h [42], while an estimate of 270 L/h was reported for a 3-day continuous infusion [43]. Population pharmacokinetic analysis performed by Etienne et al. presented an estimate of 235 L/h, where the data were described by a one-compartment model with first-order elimination [7].

BSA and C677T *MTHFR* genotype were significant covariates in our model. Despite the long-term use of BSA for 5FU dose individualization in clinical practice, existing studies provided conflicting results regarding suitability of BSA for the prediction of 5FU exposure. Some studies did not report any significant relationship between BSA and 5FU exposure [7, 37, 43], while others considered BSA as the best predictor of CL_5FU_ [5]. In a PKPD study, principally aiming to describe hematological toxicity under a combination regimen with 5FU, neither BSA nor body weight were found to influence the variability in 5FU PK [25]. Significant, but moderate, effects of either BSA [3] or body weight [44] on 5FU PK were confirmed in most population pharmacokinetic studies using nonlinear mixed-effect modelling. In the comparative covariate analysis, none of the indices representing body mass provided superiority over BSA regarding the improvement of model fit principally guided by reduction in OFV and % IIV. Patient’s gender was not found to influence CL_5FU_ in the present study, which is consistent with a previous population pharmacokinetic analysis [7]. Gender effect on CL_5FU_ observed in some studies [5, 41] might possibly be accounted for by differences in individual BSA. A considerably higher IIV of 145% was associated with *V*_c,5FU_ in comparison to previously reported values ranging from 19 to 114% [6, 37, 38, 41]. The CL_5FUH2_ (126 L/h) and *V*_C,5FUH2_ (91.9 L) estimates in our study were comparable to those reported by Mueller et al. [6]. An 18% higher CL_5FUH2_ in men was reported, but the gender influence is not supported by the present evaluation, probably because of the lower proportion of female patients in the studied population. It is worth mentioning that exploratory covariate analyses in small- to medium-sized studies are expected to result in different sets of covariates, especially in case the covariates demonstrate moderate to high correlation such as body size, age, sex, and creatinine clearance.

The effect of the *MTHFR* C677T mutation on CL_5FU_ was found to be statistically significant. Population estimates for total clearance were 278 L/h (*MTHFR* 677CT or 677CC genotype) and 150 L/h (*MTHFR* 677TT genotype), but the genotype effect was not made part of the model due to lack of mechanistic plausibility as a predictor of 5FU PK. Published studies analysed this mutation primarily in relationship with 5FU efficacy and showed its favourable role in treatment response [45] and survival [46], considering the *MTHFR* genotype as an important predictor for the therapeutic effect of 5FU [47]. The common C677T polymorphism in the *MTHFR* gene results in a considerably lower enzyme activity [46] that probably increases intracellular folate concentrations, making tumors exhibiting mutated *MTHFR* genotypes more sensitive to cytotoxicity than wild-type *MTHFR* tumors [48], if there are no differences in *MTHFR* genotype between tumor and somatic cells of the patient. It is difficult to deduce plausible mechanisms describing the influence of *MTHFR* on CL_5FU_ based on the current knowledge on the metabolic pathways which provides a motivation to investigate this effect in further studies.

The Friberg model [24] is the standard approach to study the myelotoxicity under antineoplastic treatment. The model was originally developed using total WBC count data from rats treated with 5FU. A subsequent study comprised of a number of myelosuppression models demonstrated parameter consistency across different drugs [49]. The developed models performed adequately to predict the time course of myelosuppression using both neutrophil and total leukocyte count data separately. The semi-mechanistic myelosuppression model appropriately described the total WBC count over time after 5FU administration. Transit compartments accounted for a delay between drug administration and the observed effect. Self-renewal/mitosis in the proliferating cells compartment was dependent on the number of cells, a rate constant for cell division (*k*_prol_), and a feedback mechanism from the circulating cells (Circ_0_/Circ)^*γ*^ which describes the rebound of cells as the proliferation rate is regulated by endogenous growth factors and cytokines [50]. An estimate of 6.86 × 10^9^/L for baseline leukocyte count (Circ_0_) was in the expected range [22]. The Parameter estimates for *γ* (indicative of hematopoietic viability) were highly inconsistent across model runs; therefore, the value representative of a typical population was fixed according to the available literature [22] to avoid an overshoot compared to Circ_0_. Myelosuppression was found to be significantly higher in patients receiving additional cisplatin (slope = 2.82 L/mg) as compared to the patients undergoing monotherapy (slope = 1.17 L/mg). In an attempt using a semi-physiological model to describe the relationship between the PK and the myelotoxicity contributed by respective components of the combination regimen comprised of 5FU, epirubicin and cyclophosphamide, the authors assumed negligible contribution by 5FU as it was not possible to estimate the effect contributed by 5FU and cyclophosphamide simultaneously [25]. The hypotheses underlying this strong assumption was a lower hematological toxicity observed with continuous infusions as compared to 5FU bolus administration [51], and a relatively stronger myelosuppression previously reported with epirubicin and cyclophosphamide in comparison to 5FU in rats [52]. When 5FU is investigated alone, the present results demonstrate a significant amount of myelosuppression related to 5FU continuous infusion with WBC_nadir_ values of 2.26 (× 10^9^) and 4.29 (× 10^9^) in patients receiving 5FU_comb_ and 5FU_mono_ regimens, respectively. *T*_nadir_ is typically expected between day 9 and day 14 with 5FU; however, the simulated *T*_nadir_ in the present study was observed between day 22 and day 25 after start (= 17–20 days after end) of infusion, which may possibly be attributed to the continuous nature of the infusion.

A comparative evaluation of the theoretical contribution of a 5FU dose to myelosuppression expectedly predicted a more pronounced effect for the higher dose administered in the FOLFIRINOX regimen in comparison to de Gramont regimen. Although, hematological toxicities in case of combination-based regimens are often additive in nature [52–55], a true prediction of the time course of myelosuppression under these therapeutic regimens may demand the incorporation of the effect of the other components, especially leucovorin, as one may expect differences in WBC_nadir_ and *T*_nadir_. Nevertheless, the simulations nicely show that just the FU component of even a single treatment course would put a considerable fraction of patients at risk for infections, as these doses are repeated every other week. Model-based prediction of WBC_nadir_ and *T*_nadir_ along with monitoring during the course of treatment can be imperative for suitable sampling schedules, assessment of the patient’s immune competence, and the expected consequence of additional treatment cycles [23]. Thus, it would be interesting to develop myelotoxicity models for 5-FU incorporating the effect of leucovorin in present regimens. Predictions may further be useful to identify patients or patient subgroups at a higher risk of toxicity.

## Conclusions

A semi-physiological PKPD model of 5FU is presented. IIV in the CL_5FU_ was partially explained by individual BSA. Frequent leukocyte count monitoring and model-based predictions may be used to take the contribution of 5FU to myelosuppression into account, especially in case of polychemotherapy regimens.

## Electronic supplementary material

Below is the link to the electronic supplementary material.

Supplementary file1 (PDF 969 kb)
