# Supplementary material for: Prediction of exposure-driven myelotoxicity of continuous infusion 5-fluorouracil by a semi-physiological pharmacokinetic–pharmacodynamic model in gastrointestinal cancer patients
Source: Cancer Chemother Pharmacol. 2020 Mar 9;85(4):711–22. doi: 10.1007/s00280-019-04028-5 (PMC7125253; doi:10.1007/s00280-019-04028-5)
Supplement: Supplementary file 1 — Supplementary file1 (PDF 969 kb) [file 280_2019_4028_MOESM1_ESM.pdf]

Supplementary material

Supplementary Fig. 1: Goodness of fit plots for 5FU; observed vs individual predicted (IPRED) concentration (mg/L) (A); observed vs population predicted (PRED) concentrations (B); conditional weighted residuals (CWRES) vs population predicted concentrations (C); conditional weighted residuals vs time after first dose (D). Continuous line represents the line of unity (A & B) and zero line (C & D), while dashed lines are the lines of smooth.

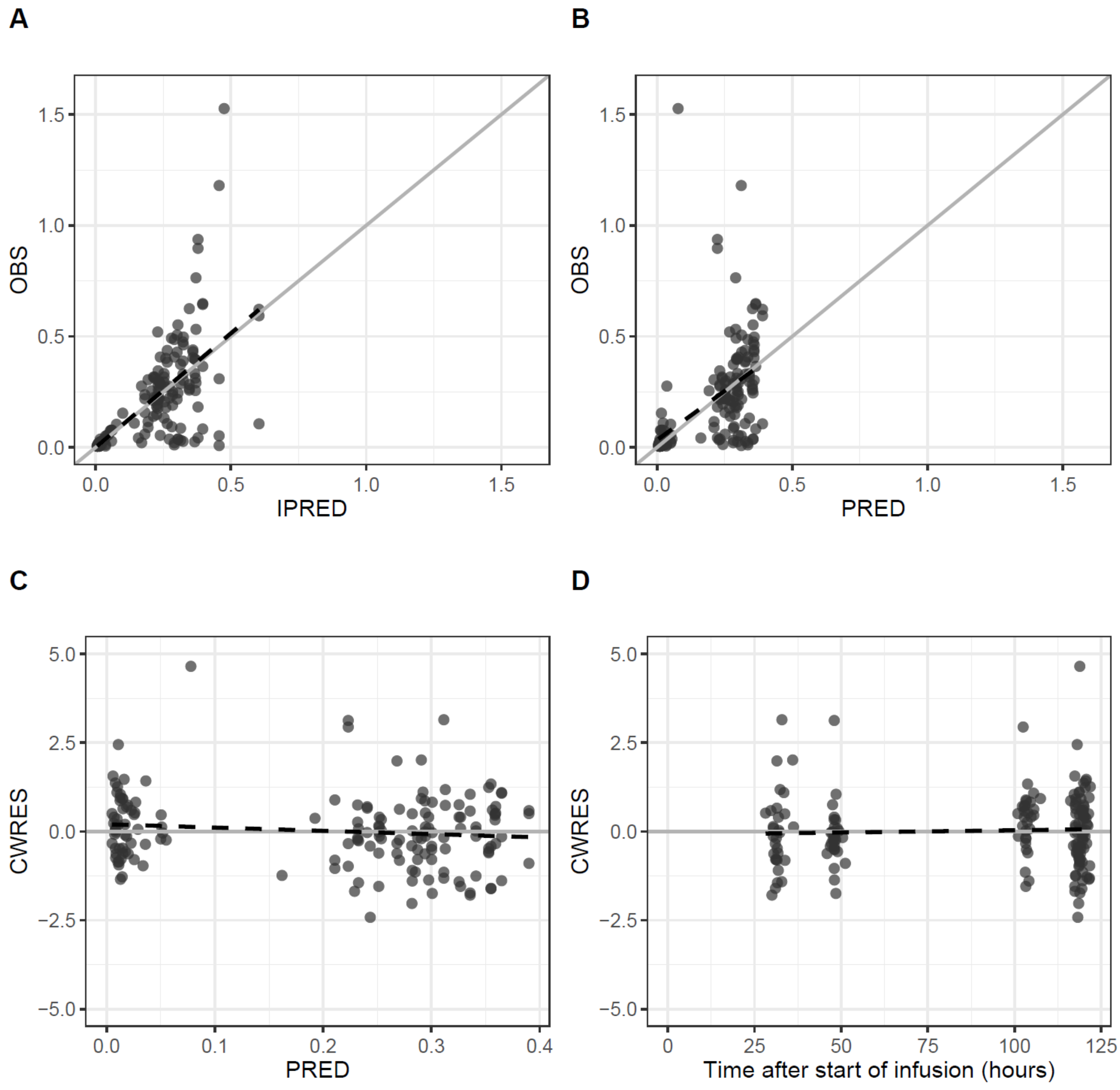

Supplementary Fig. 2: Goodness of fit plots for 5FUH2; observed vs individual predicted (IPRED) concentration (mg/L) (A); observed vs population predicted (PRED) concentrations (B); conditional weighted residuals (CWRES) vs population predicted concentrations (C); conditional weighted residuals vs time after first dose (D). Continuous line represents the line of unity (A & B) and zero line (C & D), while dashed lines are the lines of smooth.

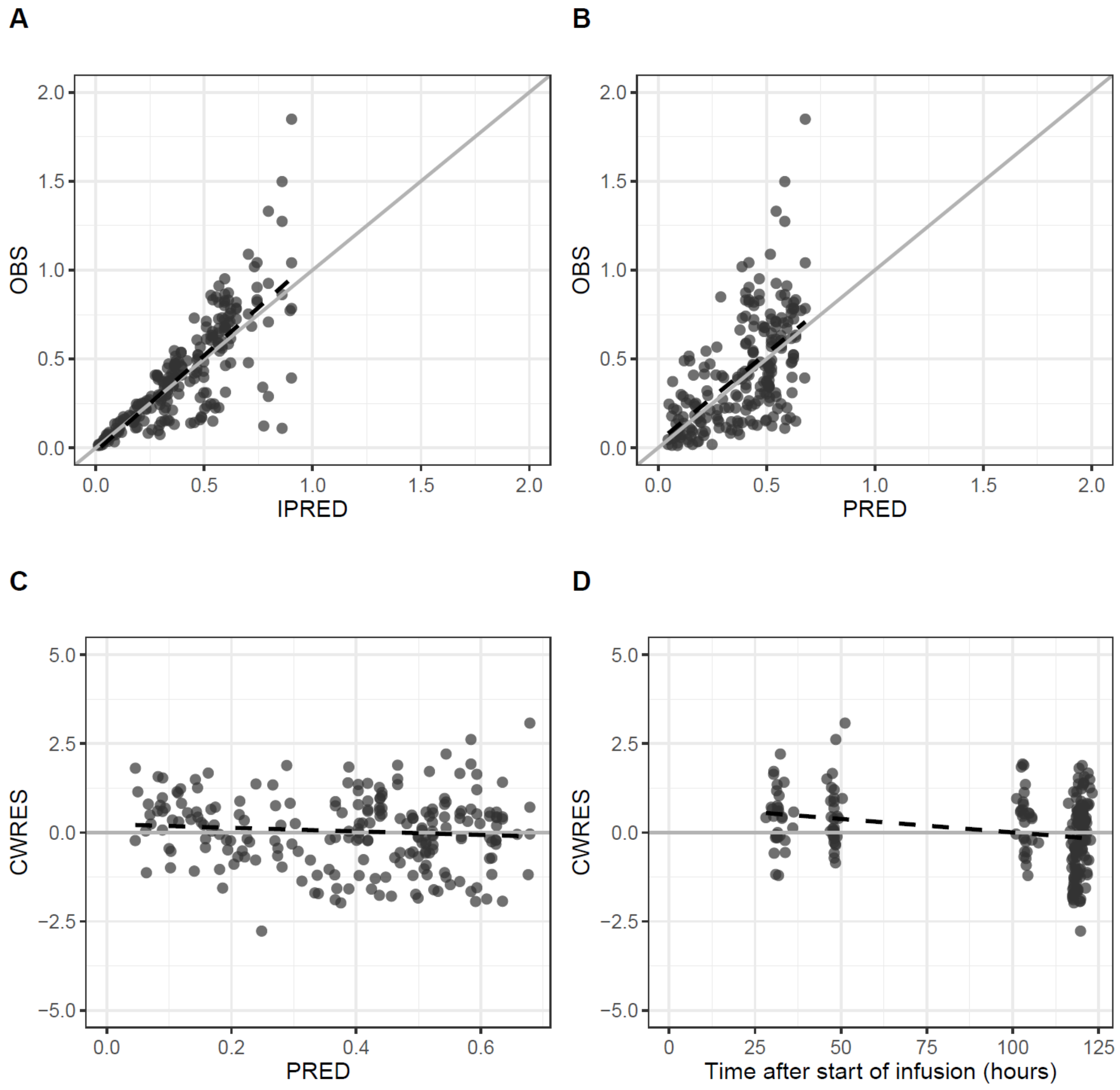

Supplementary Fig. 3: Goodness of fit plots for total WBC count data; observed (OBS)vs individual predicted (IPRED) WBC count (10^9/L) (A); observed vs population predicted (PRED) WBC count (B); conditional weighted residuals (CWRES) vs population predicted WBC count (C); conditional weighted residuals vs time after first dose (D). Continuous line represents the line of unity (A & B) and zero line (C & D), while dashed lines are the lines of smooth.

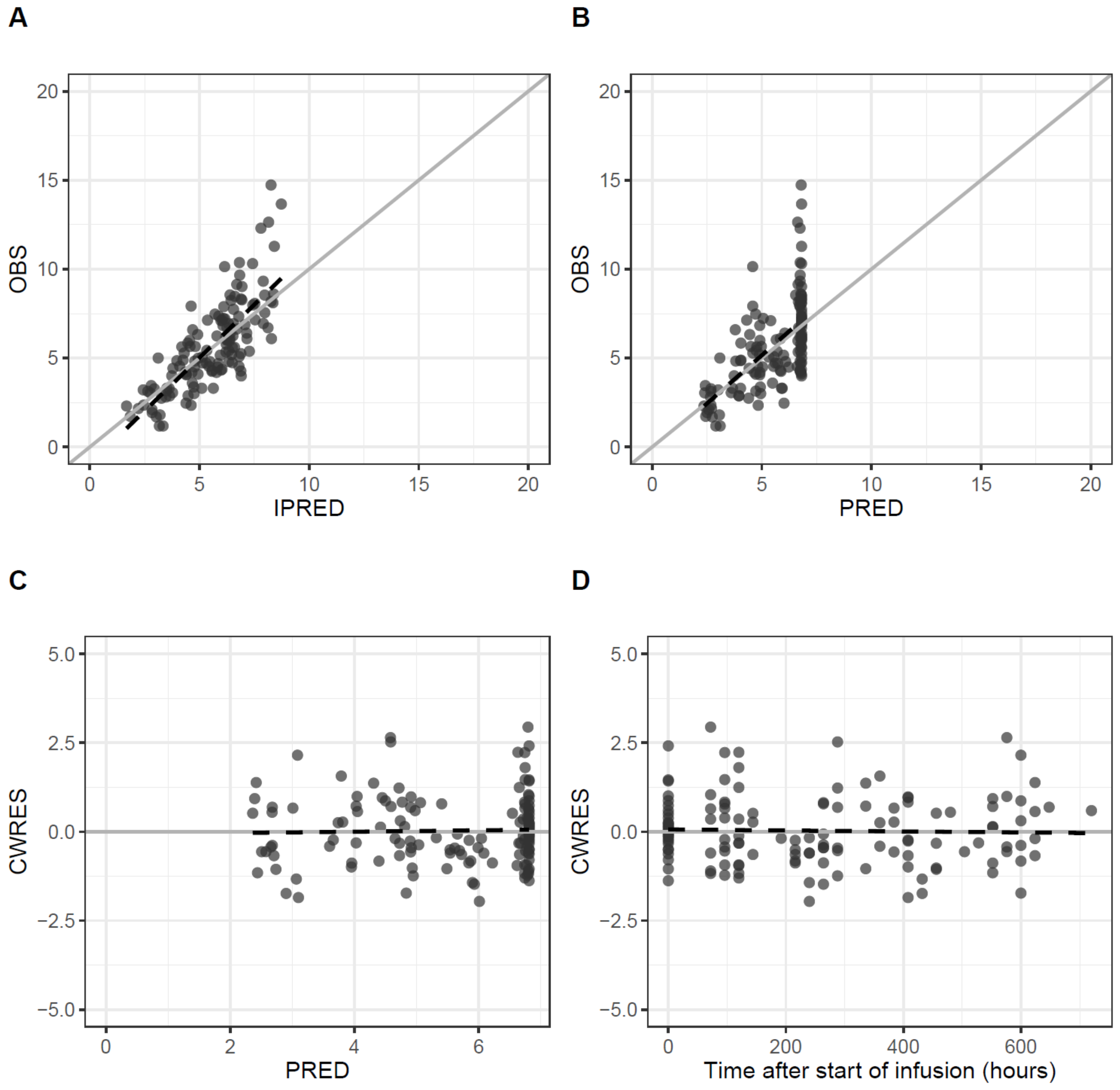

Supplementary Fig. 4: Individual plots for 5FU; points connected with dashed lines represent observed concentrations whereas continuous lines are the individual predicted concentrations.

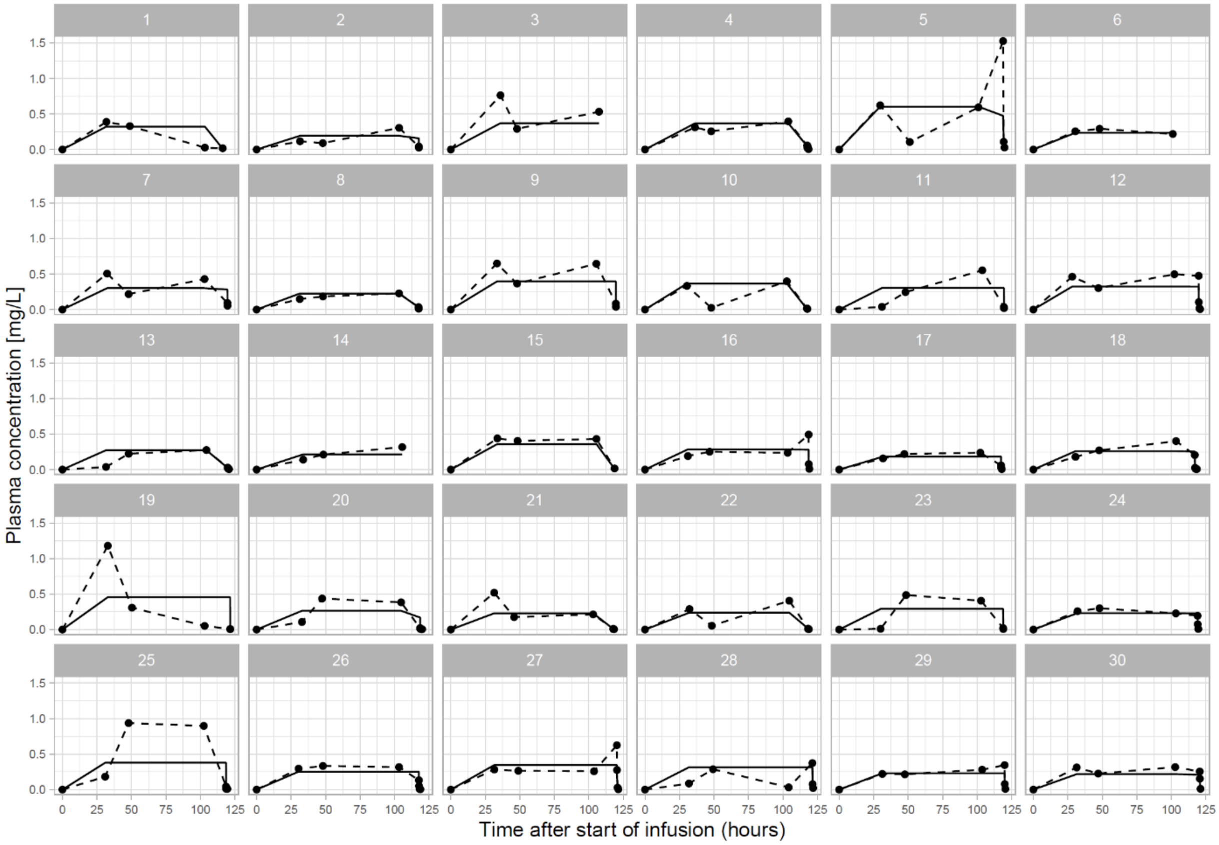

Supplementary Fig. 5: Individual plots for 5FUH2; points connected with dashed lines represent observed concentrations whereas continuous lines are the individual predicted concentrations.

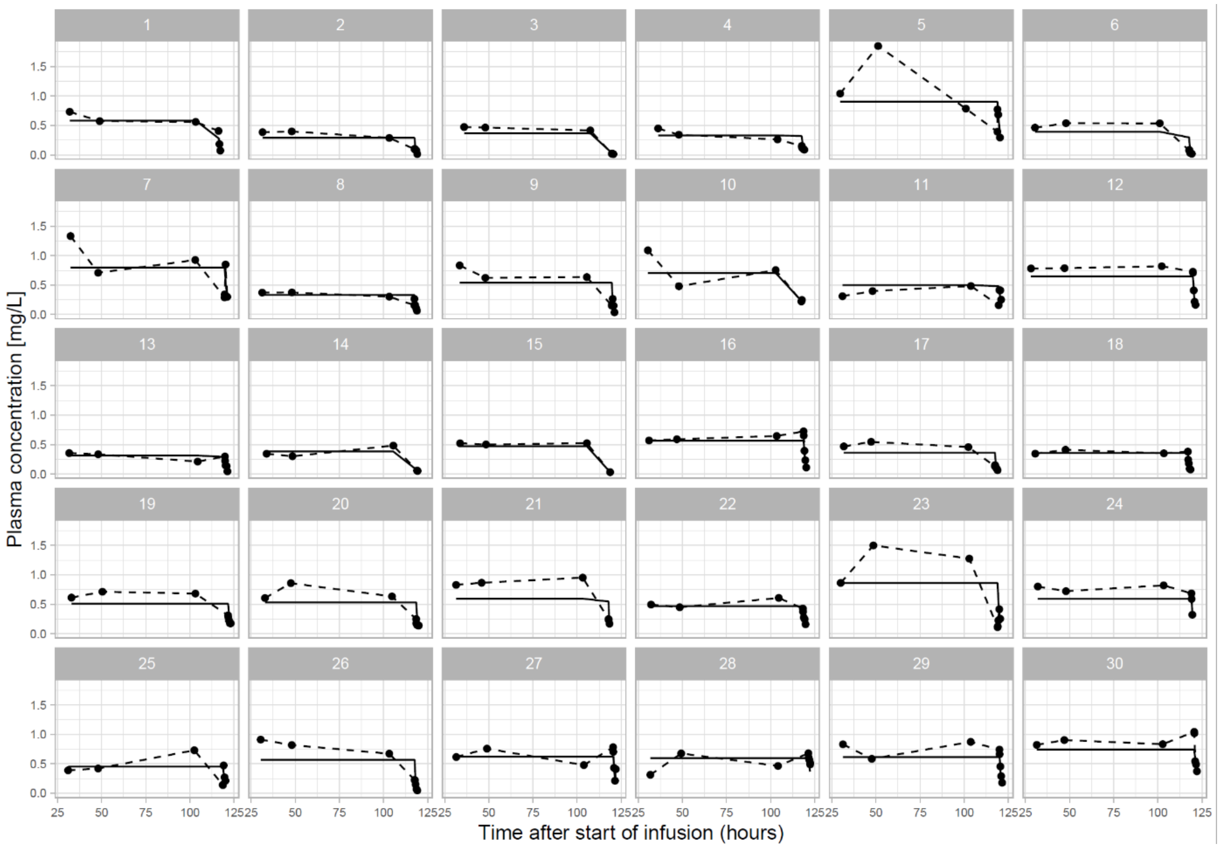

Supplementary Fig. 6: Individual plots for total WBC count data. points connected with dashed lines represent observed WBC count whereas continuous lines are the individual predicted WBC count.

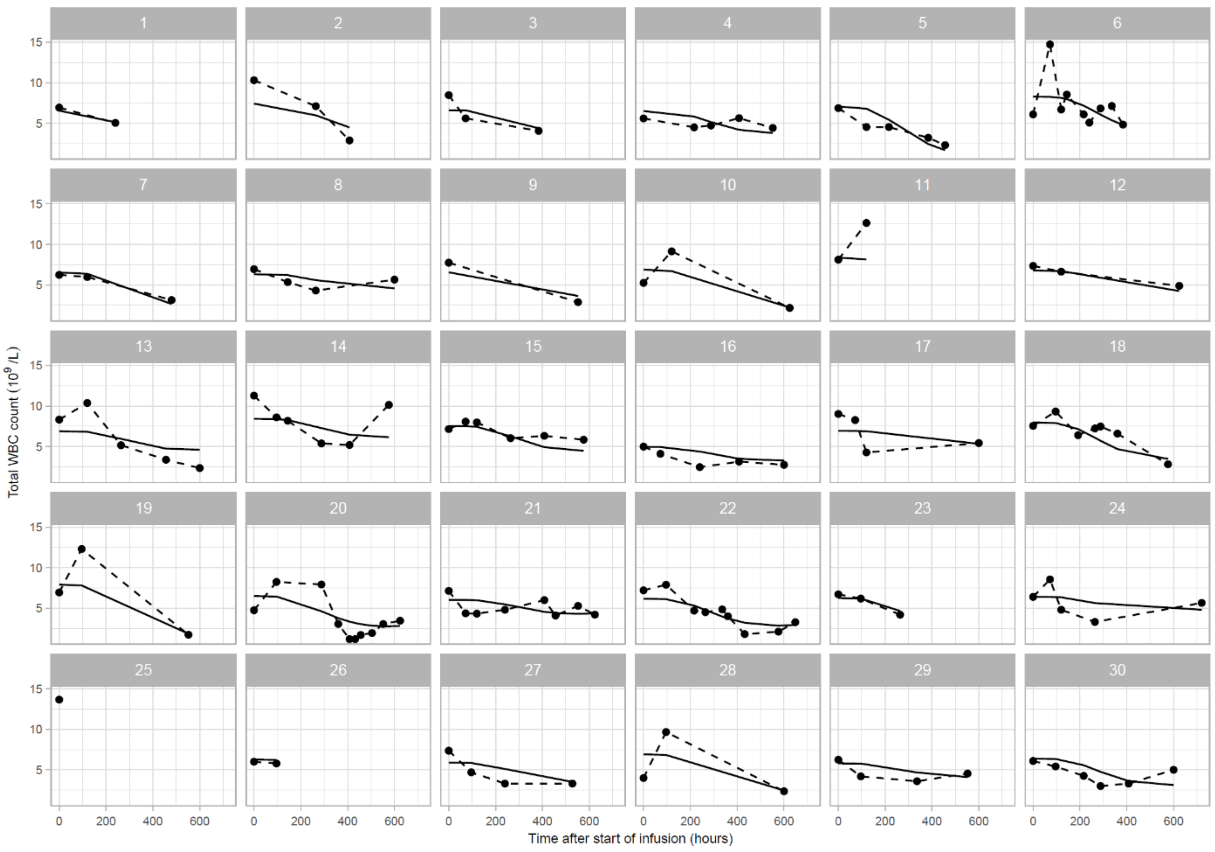

NONMEM control stream for 5FU PKPD model

```
$PROBLEM    5FU PKPD
$INPUT      ID TIME AMT RATE CMT EVID DV SE AG BS TS MR GE GT AL AS CO
            WEIGHT HEIGHT BSA BMI
$DATA       5FU.csv IGNORE=@
;-------------------------------------------------------------------
$SUBROUTINE ADVAN13 TOL=6
;-------------------------------------------------------------------
$MODEL NCOM=8
COMP = (CENT DEFDOSE NOOFF)        ; Central 5FU
COMP = (PERIP)                     ; Peripheral 5FU
COMP = (METAB NOOFF)               ; 5FUH2
COMP = (CIRC DEFOBS)               ; Circulating cells
COMP = (PROLIF)                    ; Proliferative cells
COMP = (TRANSIT1)                  ; Transit compartment 1
COMP = (TRANSIT2)                  ; Transit compartment 2
COMP = (TRANSIT3)                  ; Transit compartment 3
;-------------------------------------------------------------------
$PK
CL5FU   = THETA(1)*(1+(BSA-1.95)*THETA(8))*EXP(ETA(1))
VC5FU   = THETA(2)*EXP(ETA(2))
VP5FU   = THETA(3)
FM      = THETA(4)
CL5FU_0 = CL5FU*FM           ; CL via conversion to 5FUH2
CL5FU_1 = CL5FU*(1-FM)       ; CL of fraction not converted to 5FUH2
CL5FUH2 = THETA(5)*(1+(BSA-1.95)*THETA(8))*EXP(ETA(3))
V5FHU2  = THETA(6)*EXP(ETA(4))
Q       = THETA(7)
SLOPE   = THETA(9)
IF(CO.EQ.0) SLOPE = THETA(10)
MTT     = THETA(11)
KTR     = 4/MTT
CIRC0   = THETA(12)*EXP(ETA(5))
GAM     = THETA(13)

K12 = Q/VC5FU
K21 = Q/VP5FU
K13 = CL5FU_0/VC5FU
K31 = 0
K10 = CL5FU_1/VC5FU
K30 = CL5FUH2/V5FHU2

S1=VC5FU
S3=V5FUH2

A_0(4) = CIRC0
A_0(5) = CIRC0
A_0(6) = CIRC0
A_0(7) = CIRC0
A_0(8) = CIRC0
;-------------------------------------------------------------------
$DES
CP  = A(1)/VC5FU
```

```
CM  = A(3)/V5FUH2
EFF = SLOPE*CP
DADT(1) = - K12*A(1) + K21*A(2) - K13*A(1) - K10*A(1)
DADT(2) =   K12*A(1) - K21*A(2)
DADT(3) =   K13*A(1) - K30*A(3)
DADT(4) = - KTR*A(4) + KTR*A(8)
DADT(5) = - KTR*A(5) + KTR*A(5)*(1-EFF)*(CIRC0/A(4))**GAM
DADT(6) = - KTR*A(6) + KTR*A(5)
DADT(7) = - KTR*A(7) + KTR*A(6)
DADT(8) = - KTR*A(8) + KTR*A(7)
;-----------------------------------------------------------------------
$ERROR
CFU   = A(1)/VCFU+0.00001
CFUH = A(3)/V5FUH2
CWBC   = A(4)

IF(CMT.EQ.1) THEN
IPRED = CFU
W = THETA(14)*IPRED
ENDIF
IF (CMT.EQ.3) THEN
IPRED = CFUH
W = THETA(15)*IPRED
ENDIF
IF(CMT.EQ.4) THEN
IPRED = CWBC
W = THETA(16)*IPRED
ENDIF

IRES  = DV-IPRED
IWRES = IRES/W
Y = IPRED + EPS(1)*W
;-----------------------------------------------------------------------
$THETA
(1, 278,500)          ; CL5FU
(1, 6.52,50)          ; VCFU
(1, 33.4)             ; VP5FU
(0.85) FIX            ; FM
(10, 120,700)         ; CL5FUH2
(1, 96.7,400)         ; V5FUH2
(0.1, 16.4,100)       ; Q
(-2.63, 0.68,2.12)    ; BSA
(0, 2.95)             ; SLOPE_1
(0, 1.02)             ; SLOPE_2
(0, 269)              ; MTT
(0, 6.8)              ; CIRC0
(0, 0.17) FIX         ; GAM
(0, 0.581)            ; W_CFU
(0, 0.381)            ; W_CFUH
(0, 0.309)            ; W_CWBC
;-----------------------------------------------------------------------
$OMEGA
 0.0265               ; IIV_CL5FU
 1.04                 ; IIV_VCFU
```

```
 0.0826                ; IIV_CL5FUH2
 0.257                 ; IIV_V5FUH2
 0.0563                ; IIV_CIRC0
;-----------------------------------------------------------------------
$SIGMA 1 FIX
;-----------------------------------------------------------------------
$ESTIMATION METHOD=1 INTER PRINT=5 NOABORT MAXEVAL=9999
$COVARIANCE
;-----------------------------------------------------------------------
$TABLE ID TIME DV EVID CMT PRED IPRED IRES IWRES CWRES MR NOPRINT
ONEHEADER FILE=sdtab97
;-----------------------------------------------------------------------
```
